# The rise of high-entropy battery materials

**DOI:** 10.1038/s41467-024-45309-9

**Published:** 2024-02-01

**Authors:** Bin Ouyang, Yan Zeng

**Affiliations:** 1 Department of Chemistry and Biochemistry, Florida State University, Tallahassee, FL 32304 USA; 2 Materials Sciences Division, Lawrence Berkeley National Laboratory, Berkeley, CA 94720 USA

**Keywords:** Batteries, Batteries

## Abstract

The emergence of high-entropy materials has inspired the exploration of novel materials in diverse technologies. In electrochemical energy storage, high-entropy design has shown advantageous impacts on battery materials such as suppressing undesired short-range order, frustrating energy landscape, decreasing volumetric change and reducing the reliance on critical metals. This comment addresses the definition and potential improper use of the term “high entropy” in the context of battery materials design, highlights the unique properties of high-entropy materials in battery applications, and outlines the remaining challenges in the synthesis, characterization, and computational modeling of high-entropy battery materials.

In recent years, there has been a growing interest in high-entropy materials attributed to their remarkable physical and chemical properties observed in high-entropy alloys^1,2^ and ceramic materials^3–5^. The study of high-entropy battery materials (HEBMs) started with the development of high-entropy metal oxides as Li-ion battery anodes that exhibited improved capacity and retention^4–6^. This has then expanded to include high-entropy Li-ion cathodes, which outperformed commercialized materials in terms of energy density and rate capability^7^. More recently, high-entropy materials have also been explored for their applications as Li-ion and Na-ion solid electrolytes, showing ionic conductivities comparable to the best-performing solid-state ion conductors while offering additional benefits^8–11^.

While the progress made in the field of high-entropy materials including HEBMs is exciting, it is crucial to apply the term “high entropy” correctly and to deepen our understanding of the fundamental relationship between entropy and the enhanced properties observed. To propel the development of high-entropy materials as advantageous alternatives to traditional materials in batteries and other advanced technologies, it is imperative to make advancements in synthesis, characterization, and computational modeling. These areas of research need to be pursued vigorously to overcome existing challenges and fully exploit the potential of HEBMs.

## Definition of high-entropy materials and potential misuse in the battery field

High-entropy materials were initially proposed as single-phase materials consisting of five or more components in (near)equimolar concentrations with a substantial increase in configurational entropy^2^. This concept was originally applied to metal alloys, where alloys with an ideal configurational entropy (${S}_{{Config}}^{{Ideal}}$) equal to or greater than $1.609{k}_{{{{\rm{B}}}}}$ (${k}_{{{{\rm{B}}}}}$ being the Boltzmann constant) are categorized as “high-entropy alloys” (HEAs)^1,2,12^. Later studies have considered refractory alloys with four (near)equimolar components, associated with an ${S}_{{Config}}^{{Ideal}}$ of 1.39${k}_{{{{\rm{B}}}}}$, as refractory HEAs^13–17^.

When applying the concept of “high entropy” to HEBMs where multiple sublattices exist, the reported value of ${S}_{{Config}}^{{Ideal}}$ can be misleading if the unit is not specified. The value of ${S}_{{Config}}^{{Ideal}}$ is correlated to the normalization reference, such as by formula unit, all atoms, cations, anions, a specific sublattice, or a specific Wyckoff position^18^. For example, a Li metal oxide LiMO_2_ with a hypothetical composition Li(A_1_, A_2_, A_3_, A_4_, A_5_)O_2_, where five equimolar components are randomly distributed on the A-site, has an ${S}_{{Config}}^{{Ideal}}$ of $1.609{k}_{{{{\rm{B}}}}}$ per A-site or per formula unit, $0.805{k}_{{{{\rm{B}}}}}$ per cation, and $0.402{k}_{{{{\rm{B}}}}}$ per atom.

It is important to note that terms “high entropy”, “multicomponent”, and “compositionally complex” are not always interchangeable. Only being “multicomponent” or “compositionally complex” does not necessarily imply high entropy, as the configurational entropy relates not only to the number of mixed components but also their proportions. Under ideal mixing conditions where components are randomly distributed, the configurational entropy is calculated by

1 $${S}_{{Config}}^{{Ideal}}=-{k}_{{{{\rm{B}}}}}\mathop{\sum }\limits_{i=1}^{N}{x}_{i}{{\mathrm{ln}}}{x}_{i}$$

where ${x}_{i}$ represents the mole fraction of component *i* on the site of mixing. As an example, substituting 20% of the cation sites of a material with ten different equimolar components (each having ${x}_{i}=0.02$) would result in an ${S}_{{Config}}^{{Ideal}}$ of $0.96{k}_{{{{\rm{B}}}}}$ per cation site, calculated from

2 $${S}_{{Config}}^{{Ideal}}=-{k}_{{{{\rm{B}}}}}(10\, * \,0.02\,{{\mathrm{ln}}}\,0.02+0.8\,{{\mathrm{ln}}}\,0.8)=0.96{k}_{{{{\rm{B}}}}}$$

Although having eleven components on the cation sites, the ideal configurational entropy of this material is even lower than that of a “medium entropy” equimolar ternary alloy (${S}_{{Config}}^{{Ideal}}=1.1{k}_{{{{\rm{B}}}}}$). Note that one of the traditional layered cathode materials, LiNi_0.5_Mn_0.3_Co_0.2_O_2_ (NMC532), has an ${S}_{{Config}}^{{Ideal}}$ of 1.03 ${k}_{{{{\rm{B}}}}}$ per transition metal site. Thus, materials with multiple components but low ${S}_{{Config}}^{{Ideal}}$ should not be categorized as “high-entropy materials”^19^. Even though entropy may still play an important role in these materials, a more precise terminology would be “co-doped” or “multicomponent” materials.

Furthermore, the predominant types of entropy can vary significantly across different systems. In crystalline solid solutions, configurational entropy typically presents as the primary entropic contributor to phase stability. However, as revealed by Esters et al.^20^, vibrational entropy can also play a vital role in influencing the stability of specific high-entropy materials such as high-entropy carbides. In polymers, additional entropic factors such as conformational entropy come into play^21^. In liquid systems such as aqueous or liquid electrolytes^22–24^, entropies such as vibrational entropy, rotational entropy, translational entropy, or cavity entropy may dominate the system^25^. Therefore, for precise use of the term “high entropy”, it is important to specify the specific entropic contributors that are relevant to the particular systems being examined.

## The unique properties of HEBMs

**Fig. 1:**
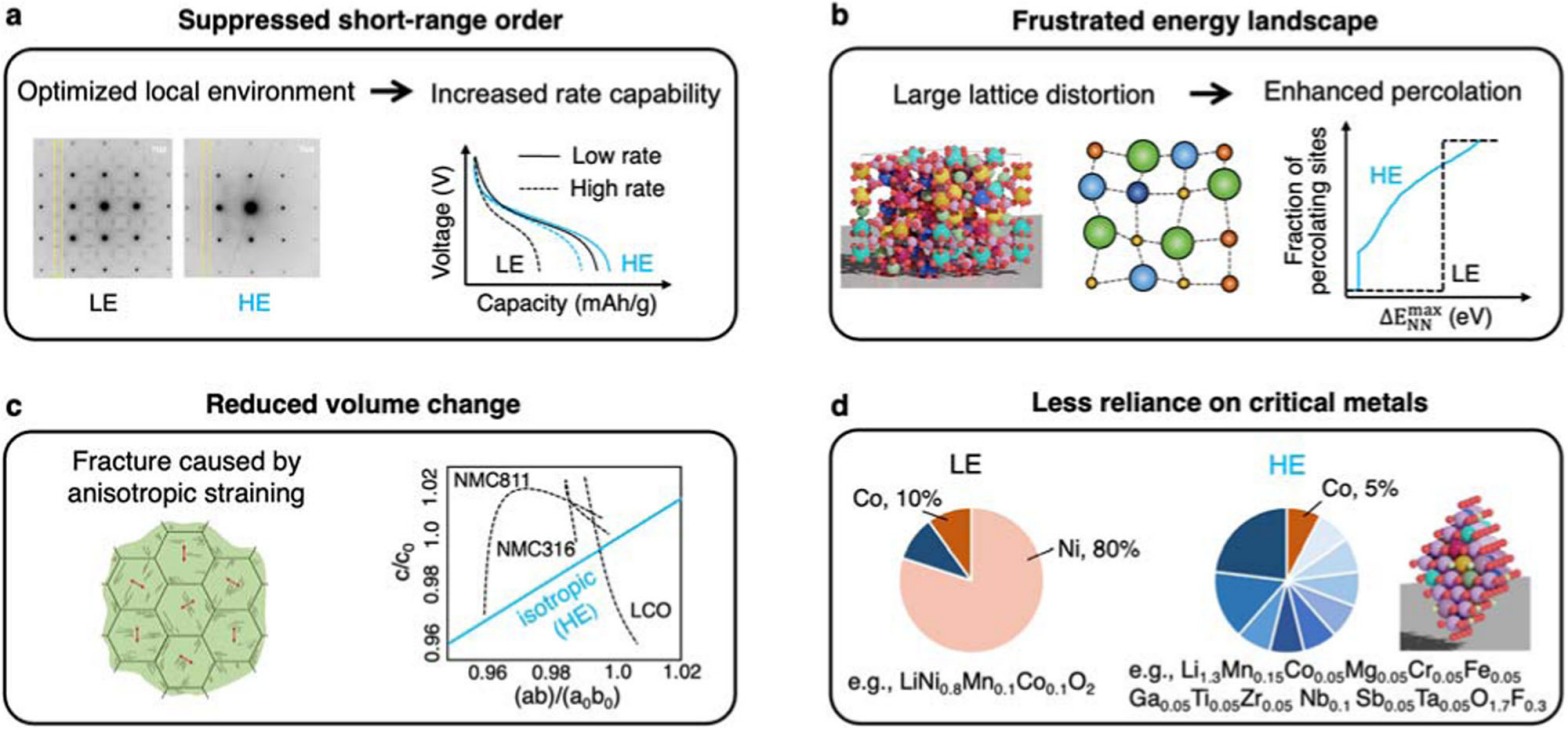
The unique properties of high-entropy battery materials (HEBMs). High-entropy (HE) materials have shown advantageous properties compared to low-entropy (LE) materials including (**a**) suppressed short-range order as shown in the diffusive scattering pattern of a high-entropy cathode material, which results in enhanced capacity and rate capability^7^ (**b**) frustrated energy landscape created by large lattice distortion and mix of diverse chemistry, leading to enhanced ion percolation observed in high-entropy ionic conductors^8^ (**c**) reduced volume change attributed to isotropic straining in contrast to anisotropic straining that causes intergranular fracture in conventional layered cathode materials^27,55^ and (**d**) reduced reliance on critical metals as compared to conventional battery materials (e.g., Ni and Co are heavily used in commercialized cathode materials). The atomic illustrations show the arrangement of diverse atoms in a Na-superionic conductor (NASICON) structure in (**b**) and a disordered rocksalt (DRX) structure in (**d**). Panel (**a**) reprinted with permission from SNCSC^7^. Panel (**b**) adapted with permission from AAAS^8^. Panel (**c**) adapted with permission from Elsevier^55^.

High-entropy materials are mostly recognized for their enhanced phase stability resulting from the high value of mixing entropy compared to traditional materials. When applied to battery applications, enhancements in tailoring short-range order, energy landscape, volumetric change, and chemical versatility make high-entropy materials particularly valuable. The identified advantages of HEBMs are summarized in Fig. 1.

The absence of principal elements in high-entropy materials can disrupt the formation of a dominant coordination environment, instead creating a diverse ensemble of local environments. Research has demonstrated that this characteristic can significantly reduce the short-range order that can harm electrochemical performance, therefore allowing HEBMs to achieve increased capacity and rate capability with cathode materials^7^. A prototype high-entropy cathode material in a disordered rocksalt structure, Li_1.3_Mn^2+^_0.1_Co^2+^_0.1_Mn^3+^_0.1_Cr^3+^_0.1_Ti_0.1_Nb_0.2_O_1.7_F_0.3_^7^, which has ${S}_{{Config}}^{{Ideal}}=1.259{k}_{{{{\rm{B}}}}}$ per cation, achieved high capacity and rate capability that outperform commercialized cathode materials^26^.

High-entropy materials also exhibit a higher tolerance of lattice distortions compared to low-entropy materials, which allows for substantial perturbation on the energy landscape for ion diffusion. Carefully engineered lattice distortions can create percolating diffusion pathways, enabling orders of magnitude increase in ionic conductivities as initially demonstrated in high-entropy oxide-based materials^8^. This phenomenon was further observed in sulfide-based argyrodites^10^.

The highly disordered nature of HEBMs also offers a novel approach to control dimensionality change in battery electrodes during charge and discharge. Many electrode materials experience significant volumetric change during the insertion and extraction of alkali metals. Researchers have found that disordering can mitigate these volumetric changes by increasing isotropicity, hence improving the capacity retention^27^. Doping with multiple components to layered structured cathode materials has also shown enhanced electrochemical performance^28^. While the use of the term “high entropy” for multicomponent-doped compounds that do not exhibit high ${S}_{{Config}}^{{Ideal}}$ is debatable, the exploration and utilization of multicomponent electrode materials underline the importance of leveraging the benefits they offer.

The improved performance of HEBMs does not rely on the presence of particular elements but rather on the collective ensemble of multiple ones. This offers opportunities for the development of more sustainable and accessible battery materials. For instance, the improved performance provided by the high-entropy disordered rocksalt cathode materials is primarily attributed to the diverse local environments that suppress short-range order^7^. Thus, any element in these materials is essentially replaceable as long as the undesired short-range orders are reduced, which is demonstrated in compositions without nickel and only 5% atomic fraction of cobalt per transition metal site, diverging from conventional battery cathodes that heavily rely on these two critical metals. This finding offers valuable insights for the development of cobalt and nickel-free cathode materials, addressing a critical concern in the electric vehicle (EV) industry^29^. In terms of solid electrolytes, similarly, the enhanced performance of high-entropy materials does not stem from specific elements but rather depends on the desired level of lattice distortion^8^. In contrast to low-entropy solid electrolyte materials that typically require elements with specific radii or electronic structures^30,31^, high-entropy materials offer more flexibility as multiple elements with distinct ionic radii can be readily utilized. This aspect highlights the potential of high-entropy materials as a design strategy to reduce reliance on critical metals in battery technologies, particularly as our understanding of the composition-structure-property relationship improves.

## Challenges and opportunities of HEBMs

### Synthesis

Synthesis of HEBMs poses significant challenges due to the vast number of possible compositions in high-dimensional composition space^32^. Conventional trial-and-error methods or brute-force enumeration are impractical for exploring such a large design space, making synthetic design difficult. Furthermore, the presence of multiple elements in high-entropy materials can lead to the coexistence of many intermediate compounds and byproducts, complicating phase identification and the understanding of reaction pathways that are critical to design target compounds and to improve synthesis procedures^33^. In the context of HEBMs, their properties depend not only on the overall compositions and the bulk structures but also on the local ordering and environments that are sensitive to experimental conditions^34^. Therefore, careful control over synthesis conditions such as heating temperature, annealing time, and cooling rate is essential for property control. Implementing advanced synthesis tools, such as high-throughput experimentation and AI-driven workflow, holds great potential to address these synthesis challenges. Autonomous laboratories that integrate robotics, high-throughput experimentation and AI agents can further benefit the development of high-entropy materials by enabling efficient iterations between synthetic design and execution in closed-loop^35,36^.

### Characterization

The advancement of HEBMs necessitates the utilization of sophisticated characterization techniques that can analyze multiple elements at low concentrations. This task becomes particularly challenging when elements exhibit similarities that hinder differentiation using conventional microscopic or spectroscopic approaches. Furthermore, the emerging design principles for high-performance HEBMs involve the distribution of local ordering and deviations in bond lengths at the atomic scale. However, characterizing these atomic-scale features remains a challenge even with the frontier instruments. Accurate analysis and in-depth understanding of HEBMs require the development of innovative characterization techniques or enhancements to existing methods. These techniques will play a crucial role in advancing our understanding of HEBMs, enabling precise control of their properties.

### Computational modeling

Computational and data science tools have provided efficient “in-silico” approaches to explore battery materials. It is important to note that challenges persist when applying these tools to high-entropy materials. In the context of high-throughput materials screening, the compositional space grows exponentially with the number of participating elements. While evaluating synthesizability for traditional low-entropy materials may require a few thousand calculations to exhaust the periodic table^37,38^, the compositional space of high-entropy materials can easily scale up to millions or billions of compositions, surpassing the capabilities of current high-throughput screening methods. Additionally, the potential competing phases for high-entropy materials increase combinatorially. While ternary oxides used in batteries typically involve competing binary oxides, elemental phases, and polymorphs^38^, HEBMs introduce a more extensive range of competing phases, including ordered phases and infinite combinations of solid-solutions with lower configurational entropy and lower mixing enthalpy.

Evaluating the synthetic accessibility of high-entropy materials necessitates considering both the configurational entropy gain that reduces free energy, and the positive mixing enthalpy, which increases it. When mixing components with substantially different size or electronic structure, the corresponding mixing enthalpy might be too excessive to be compensated by the entropy gain, making their synthesis impractical. Additionally, the actual configurational entropy will deviate from the ideal configurational entropy when the distribution of components is not fully or close to fully random, which have been seen in battery electrode or electrolyte materials in which short-range order presents^7,39,40^. The calculation of the actual configurational entropy requires more advanced statistic mechanical sampling^40,41^.

In terms of data science, the study of high-entropy materials faces challenges due to the lack of effective datasets and sufficiently accurate algorithms for prediction. Leading materials genome databases, such as the Materials Project^42^, OQMD^43^, AFlow^44^, NOMAD^45^, MARVEL^46^, contain limited data on highentropy materials (AFlow^44^ reports a dataset of highentropy carbides^20^) and no data on HEBMs. This highlights the need to establish new datasets to facilitate research on HEBMs. Moreover, the critical energy range for HEBMs typically falls within 30–60 meV/atom, which is close to or lower than the root mean square errors (RMSE) or mean absolute errors (MAE) of the most advanced global machine learning models in this field^47–49^. This narrow energy range (30–60 meV/atom) primarily arises from two factors: 1) the contribution of ${S}_{{Config}}^{{Ideal}}$ (e.g., 59.73 meV/atom for a compound with formula A_0.25_B_0.25_C_0.25_D_0.25_O at 1000 K) and 2) the energy difference between various local structures usually lies within 10–20 meV/atom (${k}_{{{{\rm{B}}}}}*300{{{\rm{K}}}}=26{{{\rm{meV}}}}/{{{\rm{atom}}}}$)^7,39,40^. These factors necessitate further advancements in building datasets and improving data science algorithms to further our understanding of HEBMs and optimize their properties.

### Expanding HEBMs applications

HEBMs have been predominately studied as Li-ion electrodes and electrolytes, with limited exploration in Na-ion systems^8,50^. The unique properties stemming from the high-entropy design hold promises for diverse battery technologies. Specifically, enhancing ion transport through controlling disordering in HEBMs can address the low ion mobility challenge in multivalent batteries such as Mg-ion and Ca-ion batteries^51–53^. Furthermore, HEBMs offer potential solutions for batteries beyond the intercalation-based systems. In Li-S and Li-air batteries, where various components necessitate distinct properties, HEBMs can contribute to enhancing ionic conductivity^8^ and catalytic performance^54^. By integrating the insights into highentropy ionic conductors^7,8^ and the recent advancements in high-entropy catalysts^54^, emerging design principles are anticipated. These principles hold the potential to drive innovation, significantly impacting the landscape of battery technologies and facilitating advancements in related scientific domains.
